# Non-islet cell tumor hypoglycemia concurrent with acromegalic features: A case report and literature review

**DOI:** 10.3389/fsurg.2022.968077

**Published:** 2022-09-22

**Authors:** Xiaojing Wang, Naishi Li, Yi Xie, Liang Zhu, Ji Li, Feng Gu, Xinhua Xiao

**Affiliations:** ^1^ Department of Endocrinology, Key Laboratory of Endocrinology of National Health Commission, Peking Union Medical College Hospital, Chinese Academy of Medical Science and Peking Union Medical College, Beijing, China; ^2^ Department of Endocrinology, Beijing Tsinghua Changgung Hospital, Tsinghua University, Beijing, China; ^3^ Department of Urology Surgery, Peking Union Medical College Hospital, Chinese Academy of Medical Science and Peking Union Medical College, Beijing, China; ^4^ Department of Radiology, Peking Union Medical College Hospital, Chinese Academy of Medical Science and Peking Union Medical College, Beijing, China; ^5^ Department of Pathology, Peking Union Medical College Hospital, Chinese Academy of Medical Science and Peking Union Medical College, Beijing, China

**Keywords:** non-islet cell tumor hypoglycemia, acromegaly, IGF-2, solitary fibrous tumor, hemangiopericytoma

## Abstract

**Background:**

Non-islet cell tumor hypoglycemia (NICTH) is a rare cause of hypoglycemia due to the overproduction of high molecular weight insulin-like growth factor (big-IGF2), which activates the insulin receptor and subsequently caused hypoglycemia. But NICTH with acromegaly had rarely been reported. We firstly reported a rare case of NICTH concurrent with acromegalic facial features induced by a retroperitoneal hemangiopericytoma and reviewed similar cases in the literature.

**Case presentation:**

A 30-year old man was admitted to hospital because of recurrent unconscious, which usually occurred in the late afternoon or early morning before supper or breakfast. On one unconscious occasion, his blood glucose was 2.4 mmol/L. His consciousness recovered rapidly with intravenous 50% glucose administration. Physical examination showed that he had coarse oily facial features with acne, prominent forehead and brow, broad nose, prominent nasolabial folds. At the time of hypoglycemia, suppressed serum insulin, GH and IGF-1 levels was found. Computed Tomography further revealed a large left retroperitoneal mass measuring 7.0 cm × 12.3 cm × 13.0 cm. He underwent complete surgical resection of the mass. Surgical pathology demonstrated a hemangiopericytoma and strong positive for IGF-2. He did not experience further episodes of hypoglycemia after the operation during the 2.5 years follow-up.

**Conclusions:**

Fibrous origin is the most common tumor type for NICTH with acromegaly features. NICTH should be considered in non-diabetic patients who have recurrent hypoglycemia along with suppressed serum insulin and IGF-1 levels.

## Introduction

Non-islet cell tumor hypoglycemia (NICTH) is a rare cause of hypoglycemia attributed to the overproduction of high molecular weight insulin-like growth factor (IGF-2), known as big-IGF2, by tumors (1). IGF-2 activates the insulin receptor, exerts insulin-like activity, and suppresses GH secretion *via* a negative feedback mechanism, resulting in hypoglycemia. In addition, IGF-2 can bind to the IGF-1 receptor, leading to acromegalic features in rare patients (1). The incidence of NICTH is estimated at one per million person-years (2). Many types of tumors have been associated with the development of NICTH (3). Although tumors of mesenchymal origin are commonly described, NICTH with acromegalic features has shown to be rare.

Here, we report a rare case of NICTH presenting with acromegaloid changes secondary to retroperitoneal hemangiopericytoma. We have also reviewed the current reports on NICTH with acromegalic features.

## Case presentation

A 30-year-old man was admitted to the outpatient Endocrinology clinic of Peking Union Medical Hospital (PUMCH) because of recurrent unconsciousness. Four months before this presentation, he had had three prior episodes of disorientation, visual changes, weakness, and palpitations in the late afternoon, and the symptoms were resolved following food intake. He did not receive any medical care. One month before admission, he was found unconscious in the early morning before eating breakfast at home and was transported by ambulance to the local hospital's emergency department. At the time of entry, his blood glucose was 2.4 mmol/L, and the patient rapidly recovered consciousness in response to intravenous 50% glucose administration. Notably, he experienced three similar episodes before this event and admission to PUMCH. He was then admitted for further evaluation and treatment following this critical information. The patient denied any history of diabetes, intake of hypoglycemic agents, and alcohol abuse. On review of his systems, he and his family noted nose enlargement, hyperhidrosis, increased acne, and coarse oily skin over approximately the last two years.

On admission, physical examination revealed a blood pressure of 146/96 mmHg, and his body mass index was 28.4 kg/m^2^. He had coarse oily facial features with acne, a prominent forehead and brow, a broad nose, prominent nasolabial folds, and acanthosis nigricans. Furthermore, multiple skin tags were present in the neck and anterior chest.

**Figure 1:**
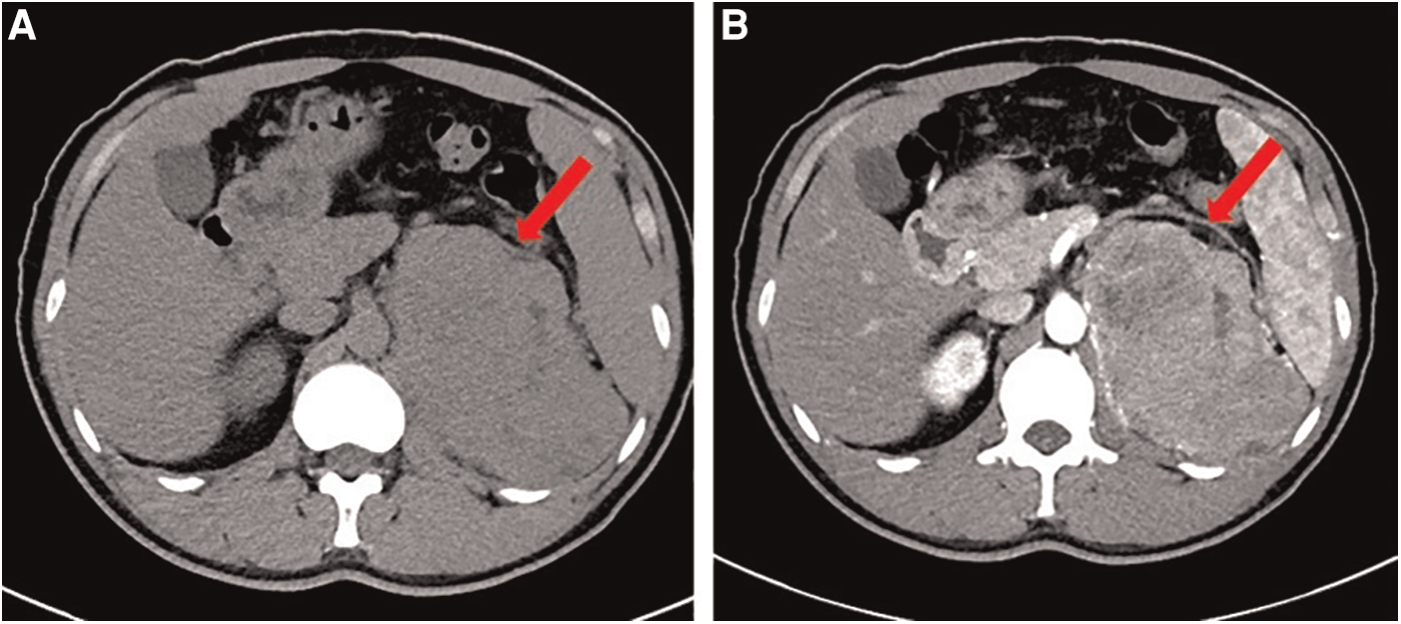
Computed tomography (CT) scan of the abdomen. (**A**). Non-Contrast CT. (**B**). Contrast CT.

Laboratory tests revealed that blood count, liver and renal function, tumor markers, thyroid function, and 24 h urinary catecholamine were all in normal ranges. The serum adrenocorticotropic and 24 h urinary free cortisol were high, but the low dose dexamethasone suppression test yielded a 24 h free cortisol of 1.76 ug. During a spontaneous morning hypoglycemia (2.2 mmol/L), the corresponding serum insulin level was less than 0.5 µIU/ml, C-peptide level was less than 0.05 ng/ml, proinsulin was 44 pg/ml. IGF-1 level was less than 25.0 ng/ml, and GH level was less than 0.05 ng/ml. The detailed laboratory findings are shown in Table 1. Based on the laboratory evaluation, the diagnoses including chronic liver disease, hypothyroidism, pheochromocytoma, adrenal insufficiency, and insulinoma were excluded, and NICTH was suspected. Contrast Computed Tomography (CT) further revealed a large left retroperitoneal mass measuring 7.0 cm × 12.3 cm × 13.0 cm with heterogeneous density, while enhanced CT scans showed moderately uneven enhancement (Figure 1).

**Table 1:** Laboratory findings on admission.

	Value	Unit	Normal range
FBG	2.2	mmol/L	3.9–6.1
C-P	<0.05	ng/ml	0.8–4.2
FINS	<0.5	ng/ml	5.2–17.2
PINS	42	pg/ml	30–180
GH	<0.05	ng/ml	<2.0
IGF-1	<25.0	ng/ml	117–329
ALT	27	U/L	9–50
AST	20	U/L	15–40
Cre	58	µmol/L	45–84
TC	4.03	mmol/L	2.85–5.70
TG	0.68	mmol/L	0.45–1.70
HDL-c	1.67	mmol/L	0.93–1.81
LDL-c	1.88	mmol/L	<3.37
Cortisol (8:AM)	20.04	µg/dl	4.0–22.3
ACTH (8:AM)	92	pg/ml	0–46
24 hUFC	242.06	µg	12.3–103.5
24 h urine DA	300	µg	120.93–330.59
24 h urine E	2.25	µg	1.74–6.42
24 h urine NE	37.5	µg	16.69–40.65
Free T3	4.07	pg/ml	1.80–4.10
Free T4	1.21	ng/dl	0.81–1.89
TSH	1.568	µIU/ml	0.38–4.34

FBG, fasting blood glucose; C-P, C-peptide; FINS, fasting insulin; PINS, proinsulin; GH, growth hormone; IGF-1, insulin-like growth factor-1; ALT, alanine aminotransferase; AST, aspartate aminotransferase; TC, total cholesterol; TG, triglycerides; HDL, high density lipoprotein; LDL, low density lipoprotein; DA, dopamine; E, adrenaline; NE, Norepinephrine; ACTH, adrenocorticotrophic hormone; 24 hUFC, 24 h urinary free cortisol; FT3, free triiodothyronine; FT4, free thyroxine; TSH, thyroid stimulating hormone.

**Figure 2:**
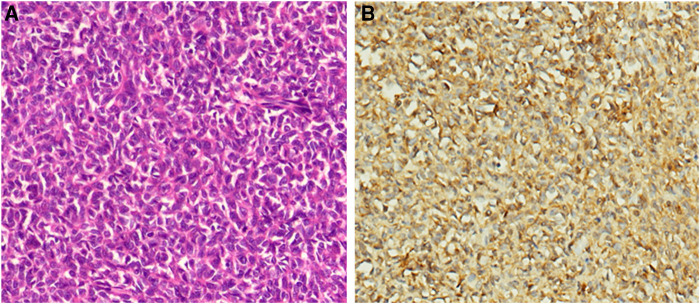
Pathological findings of the surgically resected tumors. (**A**). Hematoxylin-eosin (HE) staining (×150). (**B**). Immunostaining for IGF-2 is diffuse positive (×150).

Following these assessments, laparotomy was performed. Intraoperatively, an about 12 cm * 12 cm tumor with obvious varicose veins was found in the retroperitoneum, and the tumor adheres to the surrounding tissues. There was no visiable metastasis. After carefully sepatating the tumor, the patient underwent complete surgical resection of the mass (Supplementary Figure S1). Surgical pathology demonstrated a hemangiopericytoma and a strong positive for IGF-2 (Figure 2). The ki-67 index was 15% (Supplementary Figure S2). He did not experience subsequent episodes of hypoglycemia after the operation. A CT scan of the patient's abdomen was conducted for monitoring in our hospital every six months, and a 2.5-year follow-up did not demonstrate any evidence of recurrence.

## Discussion

In 1988, Daughaday et al. reported the first case of NICTH due to IGF-2 tumor hypersecretion by thoracic leiomyosarcoma, and the recurrent severe hypoglycemia resolved after tumor resection (3). Since then, more than 200 clinical cases of NICTH have been described. It is now recognized that a comprehensive range of IGF-2-secreting tumor types is associated with hypoglycemia. Tumors of epithelial or mesenchymal origin are commonly reported, and the predominant etiology are hepatocellular carcinomas and fibrosarcomas, respectively (1). This is the first published case of NICTH with acromegalic features induced by retroperitoneal hemangiopericytoma, to the best of our knowledge.

The *IGF-2* gene, near the *INS* gene, is located on chromosome 11p15.5 and translated into the pre-pro-IGF-2 peptide, which is sequentially processed to pro-IGF2 and the 67-amino-acid mature IGF-2 (4). Typically, approximately 80% of IGF-2 is bound to IGFBP-3 and acid-labile subunit (ALS) in the circulation, forming a ternary 150-kDa complex. About 20% of IGF-2 is in a 50-kDa binary complex containing IGFBP-3 and IGF-2 (5). In IGF-2 secreting tumors, the increased IGF-2 mRNA expression produces a more considerable amount of pre-pro-IGF-2, leading to incomplete processing of pro-IGF-2 (known as big IGF-2) (6). The excessive big IGF-2 interfered with binding the pro-IGF-2-IGFBP-3 complex to ALS, and the proportion of ternary to binary complexes is reversed, with 20% ternary and 80% binary. Low levels of ALS and IGFBP-3 magnify the impaired binding of big-IGF-2 (7). The binary complexes can cross the capillary membrane and act on insulin receptors in most tissues, which causes hypoglycemia (8). In addition, the suppressed GH and IGF-1 levels mediated by the negative feedback of increased IGF-2 *via* the IGF-1R in the hypothalamus also contribute to the hypoglycemic effects of IGF-2-omas. At present, a widely available assay for assessing big-IGF-2 is scarce. Although normal IGF-2 levels are frequently reported in NICTH (9), the IGF-2 to IGF-1 ratio is elevated, and the ratio exceeding 10:1 has been considered an important screening tool for NICTH (1, 10). In our case, the serum level of IGF-2 was not measured, but immunohistochemistry exhibited that the tumor cells highly expressed IGF-2. Regardless, undetectable IGF-1 and GH, along with suppressed insulin and C-peptide, strongly suggested hypoglycemia induced by IGF-2-producing tumor.

In addition to the hypoglycemia effect of IGF-2, growth-promoting changes have been described in rare instances of NICTH. In the present case, coarse acromegalic facial features were observed, which are thought to be mainly ascribed to IGF-2 activation of multiple subclasses of insulin-related and IGF-1 related receptors (1). We reviewed the literature on NICTH accompanied by acromegaliod face changes, and nine case reports were identified (11–17), see Table 2. The average age was 60 years (89% of patients above 50 years), with 5 females and 4 males. The most common tumor types were of mesenchymal origin, in line with previous observations. Fibrous tissue tumors (6 cases) were the most frequent among them. Pathologically, hemangiopericytoma is extremely similar to solitary fibrous tumors, but to the best of our knowledge, Hypoglycemia with acromegalic facial induced by hemangiopericytoma has not been reported. The tumors associated with NICTH are generally slow-growing and commonly quite large at the time of diagnosis (3). Fukuda et al. reported that 70% were larger than 10 cm in diameter (10). Hypoglycemia is usually the first presenting syndrome, which facilitates the identification of the tumors. In the present reviewed case series, the tumor diameter was greater than 10 cm in all patients, similar to our case. Besides, all patients had a ratio of IGF-2 to IGF-1 of more than 3, and five patients had a ratio greater than 10.

**Table 2:** Reviewed current case reports on NICTH with acromegaloid changes.

Year	Age	Sex	Tumor location	Histopathology	Tumor size	IGF-2:IGF-1	Intervention
2019 (11)	60	Male	Liver	Solitary fibrous tumors	6 × 13 × 11 cm	8.54	Portal embolization + sugery + prednisone
2019 (12)	69	Femal	Pleural	Solitary fibrous tumors	17.5 cm	20.4	Sugery + prednisone + glucagon
2019 (12)	70	Femal	Pleural	Solitary fibrous tumors	21 cm	31.8	Sugery + prednisone
2013 (13)	77	Male	Retroperitoneal mas	Fibroma	16 × 16 cm	23.6	Sugery + chemotherapy + RT + prednisolone
2013 (13)	32	Femal	Right suprarenal	Adrenocortical carcinoma	15.3 × 12.7 × 12 cm	8.6	Sugery
2011 (14)	53	Femal	Right adrenal gland	Phaeochromocytoma	15 × 14 × 20 cm	31.5	Octreotide + diazoxide
2006 (15)	53	Male	Liver	Solitary fibrous tumors	NA	NA	Sugery
2000 (16)	63	Femal	Left lung	Pleural fibrosarcoma	18 × 10 cm	11.2	Sugery
1995 (17)	60	Male	Pelvic tumor	Clear cell sarcoma	NA	3.3	Sugery

NA, not available; RT, radiation therapy.

Regardless of tumor types, surgical resection is the mainstay of therapy for IGF-2-oma. Many case reports have frequently demonstrated resolution of hypoglycemia after complete resection (1). When total resection is impossible, debulking followed by chemotherapy and radiation (depending on tumor pathology) can be considered. These adjuvant treatments have also been reported to successfully ameliorate hypoglycemia (18). Except for tumor-directed therapies, glucocorticoids have been described as the most effective drugs for NICTH and are used as a “bridge” therapy to resection (19). In our reviewed case series, 4 cases included glucocorticoid treatment. One of the underlying mechanisms that glucocorticoids undertake to prevent hypoglycemia is suppressing the production of big-IGF-2 in a dose-dependent manner. Other agents such as rhGH, somatostatin analogs, and diazoxide have also been reported in a few cases (1).

In conclusion, we present a rare case of NICTH concurrent with acromegalic facial features induced by a retroperitoneal hemangiopericytoma. Data from literature indicate that fibrous origin is the most common tumor type for NICTH with acromegaly. NICTH should be considered in non-diabetic patients with recurrent hypoglycemia and suppressed serum insulin and IGF-1 levels. Earlier diagnosis of NICTH will help to completely resect the underlying tumor more successfully.

## Data Availability

The original contributions presented in the study are included in the article/**Supplementary Material**, further inquiries can be directed to the corresponding author/s.

## Supplementary material

Supplementary Figure 1: Macroscopic view of the resected tumor.

Supplementary Figure 2: Immunostaining for Ki-67 (×150).
