# Supplementary figures and images for: Non-islet cell tumor hypoglycemia concurrent with acromegalic features: A case report and literature review
Source: Front Surg. 2022 Sep 22;9:968077. doi: 10.3389/fsurg.2022.968077 (PMC9540468; doi:10.3389/fsurg.2022.968077)

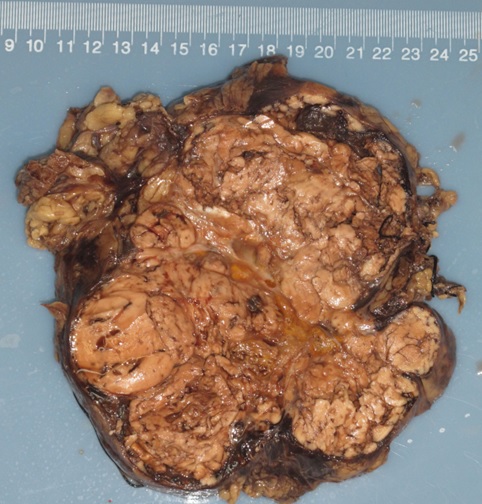

Supplement: Supplementary Figure 1: Macroscopic view of the resected tumor. [file Image1.jpeg]

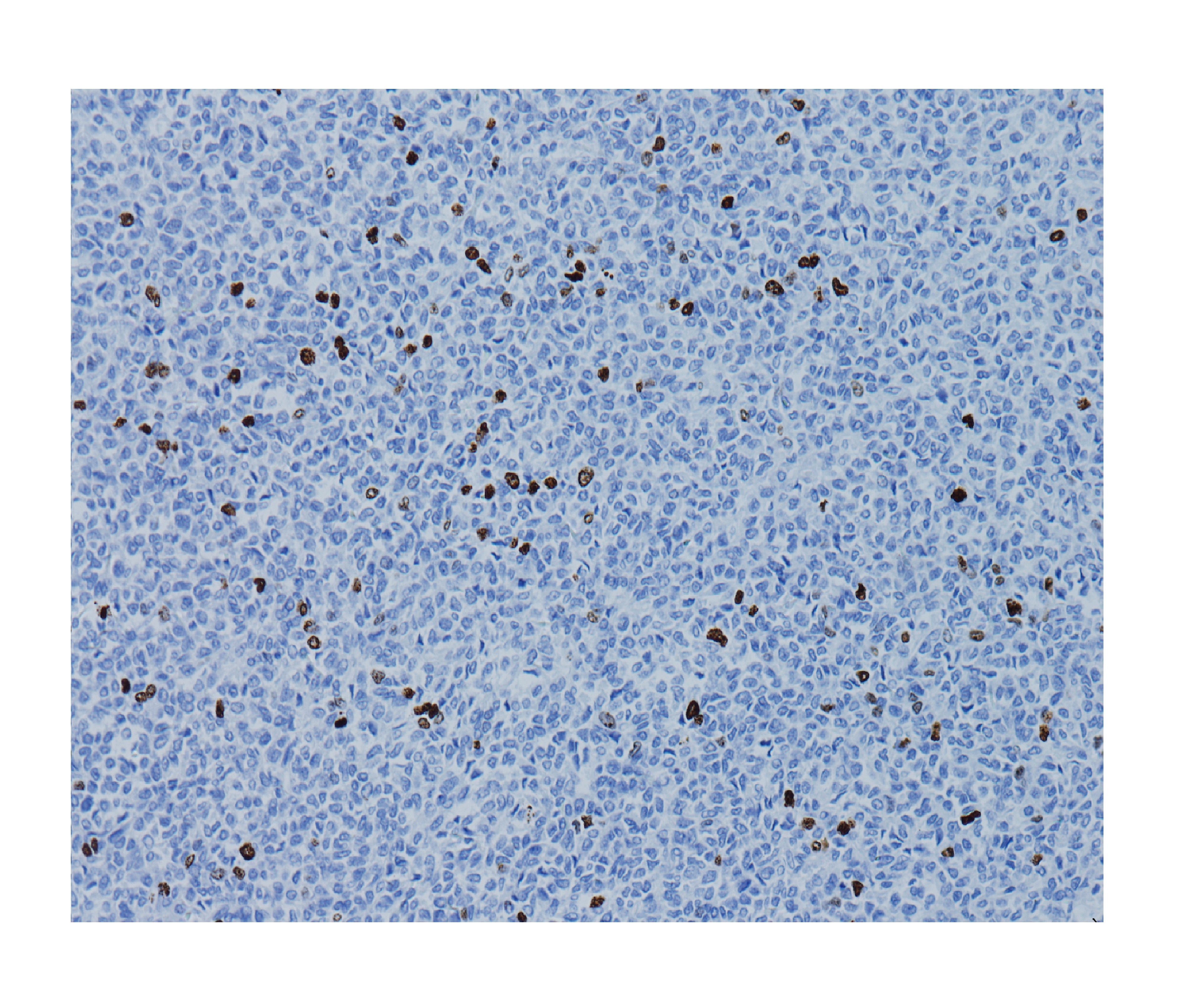

Supplement: Supplementary Figure 2: Immunostaining for Ki-67 (×150). [file Image2.jpeg]
